# Error and Bias in Geocoding School and Students’ Home Addresses

**DOI:** 10.1289/ehp.11542

**Published:** 2008-08

**Authors:** Eric A. Whitsel

**Affiliations:** Department of Epidemiology, School of Public Health, University of North Carolina, Chapel Hill, Chapel Hill, North Carolina, E-mail: ewhitsel@email.unc.edu

Zandbergen and Green (2007) recently described the effect of positional error on the distance between geocoded addresses and major roads, an often-used proxy for traffic-related exposures. They found a 200–500 m range of mean positional errors in their study of 126 Orange County, Florida, public school addresses, a somewhat higher range than that associated with geocodes assigned by four commercial vendors to a larger variety and number of street addresses in the 48 contiguous U.S. states (Whitsel et al. 2006). In both studies, however, the ranges exceeded commonly used thresholds for identifying those at greatest potential risk of traffic-related exposures, raising due cause for concern.

Zandbergen (2007) found that the use of such low thresholds to define traffic-related exposure surrogates leads to the consistent overestimation of the number of Orange County school children at risk. In this recent study (Zandbergen and Green 2007), the finding has been extended to the schools the children attend. To explain the overestimates, Zandbergen and Green illustrated the idiosyncratic positioning of schools and homes—both within land parcels and along street segments—and the uniformly higher percentage of false positive versus negative determinations of whether the geocoded locations were inside or outside the 50–1,000-m buffer radii examined in their studies.

The collective findings of Zandbergen and Green (2007) nonetheless differ from those based on a previously described 5% random sample of 2,608 street addresses from the Environmental Epidemiology of Arrhythmogenesis in WHI (EEAWHI) (Whitsel et al. 2006). In that study, we found that the fraction of participants’ addresses determined to be < 100 m from the nearest highway was relatively constant across mean positional errors of 150–600 m, a finding driven by the counterbalance of approximately equal false positive and negative rates over the same range. The sensitivity and specificity of the 100-m threshold tested in EEAWHI—one-fifth the minimum distance to schools deemed acceptable by Zandbergen and Green—were also around 90% at positional errors of 250–300 m. Moreover, even when the sensitivity and specificity of the 100-m threshold exceeded 90%, its strength of association with coronary heart disease was still underestimated, albeit in the absence of confounding and under the assumption of nondifferential misclassification.

It is tempting to generalize about the magnitude of error and direction of bias observed by Zanbergen and Green (2007)—to students’ school and home addresses outside Orange County, or more generally to epidemiologic measures of environmental exposure–health outcome association—but the most prudent course of action may be to wait until the external validity of their potentially important findings is established.
